# Presenteeism and social interaction in the “new normal” in Japan: a longitudinal questionnaire study

**DOI:** 10.1265/ehpm.23-00201

**Published:** 2024-01-20

**Authors:** Megumi Yoshigai, Jung-ho Shin, Hiroyuki Nagano, Takayo Nakabe, Yuichi Imanaka, KU Task Force to Create Resilient Societies with and after COVID-19

**Affiliations:** 1 Department of Healthcare Economics and Quality Management, Graduate School of Medicine, Kyoto University, Kyoto City, Japan; 2 The Database Center of the National University Hospitals, The University of Tokyo Hospital

**Keywords:** Presenteeism, COVID-19 pandemic, Social interaction, Social support

## Abstract

**Background:**

Although social interaction and social support during the “new normal” due to coronavirus disease 2019 (COVID-19) may be related to presenteeism, the effect between these factors has not been clear for Japanese workers. The aim of this study was to describe the presenteeism of Japanese workers with reference to social interaction and social support following the lifestyle changes due to COVID-19 and to assess whether social interaction and social support affected their presenteeism.

**Methods:**

The data were obtained from internet panel surveys from October 2020. Descriptive statistics were calculated, and multiple linear regression was conducted using the data from the first, fourth and fifth surveys, which were conducted during October to November 2020, July to August 2021, and September to October 2021, respectively. To measure presenteeism, questions from “absenteeism and presenteeism questions of the World Health Organization’s Heath and Work Performance Questionnaire”, short version in Japanese was utilized. Multiple linear regressions were conducted to investigate the effects of social interaction and social support-related factors on presenteeism.

**Results:**

A total of 3,407 participants were included in the analysis. The mean score of absolute presenteeism from the fifth survey was 58.07 (SD = 19.71). More time spent talking with family, a larger number of social supporters and a higher satisfaction level for social support were associated with a higher absolute presenteeism score.

**Conclusions:**

Our results suggested that social support reduced the presenteeism of the Japanese workers during the “new normal” due to the COVID-19 pandemic. Social interaction with family also relieved presenteeism.

**Supplementary information:**

The online version contains supplementary material available at https://doi.org/10.1265/ehpm.23-00201.

## Background

The coronavirus disease 2019 (COVID-19) outbreak was declared as the global pandemic by the World Health Organization on 11^th^ March 2020. In Japan, the first state of emergency was declared in seven prefectures on 7^th^ April 2020, which was later expanded to all prefectures on April 16^th^, 2020. With the declaration, the Japanese government requested to follow the restriction measures such as “stay-at-home”, and “suspension of business” (shorten operation hours). The “stay-at-home” measure in Japan was limited to a “request” without any legal obligation, thus differing from so-called “lockdowns” implemented in the United States or United Kingdom. However, it was reported that outings fell by 8% in prefectures with a state of emergency declaration, which resembled the percentage of decreased visits to stores during lockdowns in the United States [1]. This may indicate that even less severe restriction compared to the other countries had an impact in Japan. Furthermore, the “Atarashī Seikatsu Yōshiki (new lifestyle)” has been promoted upon lifting the first state of emergency [2]. Atarashī Seikatsu Yōshiki is commonly referred to as the “new normal” in other countries. Avoiding gathering or chatting in public, working remotely, and keeping physical distances were encouraged in this promotion. Even though this promotion was even much milder than state of emergency, it is widely acknowledged that there were certain social expectations for self-restrain, which could limit the social interaction [3]. See the supplementary table (Table S1) for the examples of practicing the “new normal” in Japan [2].

As the “new normal” permeated through both our private and professional lives, potential challenges to increase presenteeism were also pointed out along with adaptation to the social restrictions and remote work [4, 5]. Presenteeism is generally defined as the condition of attending work when one is unwell [6] or a poor job performance [7, 8]. We use the term, presenteeism, to describe inadequate work performance [8] in this study. Although absenteeism is more visible due to the physical absence of employees, presenteeism is reported not only much more common, but also more damaging for individuals and organizations [4]. Physical and mental health, lifestyle [9], financial situation, family-work/work-family conflict [10], support from supervisors [11], and personality [12] are reported as personal factors that are related to presenteeism.

In the context of COVID-19 pandemic, Kinman and Grant reported that pandemic and its consequences may intensify risk factors of presenteeism [4]. With the increasing trend of remote working, the rising concerns over work-family conflict were reported [13, 14]. According to reports in Japan, the time spent with family had increased for some Japanese during the pandemic [15]. In a report from Nikkei, the longer time spent at home and the social stress of the changed lifestyle were highlighted as potential causes behind the higher risk for family conflict and rising stress at home [16–18]. In contrast to the increase in time spent with family, the “new normal” limited the opportunities for social bonding and hindered functional social support, potentially leading the individuals to social isolation [18]. Social isolation can be defined by a lack of social interaction, receipt of social support, provision of social support, and social participation [19]. It was reported that the prevalence of anxiety and depressive symptoms were higher in lonely individuals [20], which could result in presenteeism [21]. As reported in previous study, emotional exhaustion, stress, and family-work/work-family conflict were related to presenteeism [10], whereas social interaction and support could confer protections for workers [11, 22]. Furthermore, it was reported that social restrictions increase not only the risk of developing psychopathologies but also to impair immune system [23]. Because issues in mental and physical health have been known as risk factors for presenteeism, it is reasonable to contend that social interaction and social support in the changed lifestyle may be related to presenteeism or the ability to maintain work performance. However, the presenteeism in relation to social interaction and social support in the “new normal” in Japan is not known.

Therefore, the first aim of this study was to describe the presenteeism of Japanese workers with reference to social interaction and social support following the COVID-19 pandemic. The second aim was to assess whether social interaction and social support affected the presenteeism.

## Methods

### Data source

The data were obtained from internet panel surveys conducted as a part of “research to propose a policy framework for sustainable healthcare and socioeconomic systems resilient to the COVID-19 crisis” [24]. The internet panel surveys have been repeatedly conducted from October 2020 utilizing the service provided by a Japanese survey company Rakuten Insight, Inc., which holds 2,200,000 people in the panel [25]. Role of Rakuten Insight, Inc. was to provide support for formation, distribution, and collection of our surveys. Participants were recruited via email or push notification from apps, where the priority was given to those who had accessed to the Rakuten website more than once a week. They aimed to collect at least 10,000 responses from each survey, utilizing stratified sampling by sex, 10-year age groups, and 47 prefectures of residence with the awareness that our sample does not represent the general population in Japan due to the nature of Internet panel survey. From the second survey, the individuals who had participated in the preceding survey were prioritized to answer the following surveys. Then, new participants were recruited to complement the number of dropouts. The surveys included questions about socio-economic status, personality traits, physical and mental health status, social support, changes in lifestyle and work. In the repeated surveys, some questions were added or omitted. For example, the questions about personal traits, which are less likely to change in a few months, were asked only in the first survey.

### Study population

This study used the data from the first, fourth and fifth surveys, which were conducted from October to November 2020, July to August 2021, and September to October 2021, respectively. See supplementary Fig. S1 for the timing of the surveys and number of new COVID-19 cases in Japan. The timing of the first survey was between 4 and 5 months after the first state of emergency was lifted on 25^th^ May 2020 for all prefectures. The fourth survey took place when the status of emergency was expanding to numerous prefectures in July 2021 as the number of COVID-19 cases was increasing. The fifth survey was conducted from September to October 2021, which was soon after the state of emergency was lifted on 28^th^ September for all prefectures. The fourth survey was used here because questions related to this study were included from the fourth. To ensure that the outcome variable occurred chronologically after the time point of the exposure variables, the outcome variables were utilized from the fifth survey throughout all models. The exposure variables were from the first and the fourth. The first survey was utilized for the variables of personality, where related questions were asked only in this survey. As the study population, workers aged between 20 and 69 years and who answered the first, fourth and fifth surveys in the period of October 2020 to October 2021 were included.

### Statistical analysis

Descriptive statistics summarized the participants’ demographics, distribution of absolute presenteeism score, and absolute presenteeism score by each factor representing social interaction and social support; change in time spent talking with family, change in time spent talking with friends, living with others, number of supporters, and satisfaction levels for the social support. To investigate the effects of social interaction and social support-related factors on presenteeism, multiple linear regression was applied. In total, five separate models were fitted for each exposure variable: models one to three for social interaction, and models four and five for social support.

### Outcomes

Absolute presenteeism score was the outcome of interest following the example of former studies that used absolute score rather than relative score to assess presenteeism [26, 27]. Due to the imperative of limiting the number of questions in our comprehensive survey for sustainable healthcare and socioeconomic systems resilient to the COVID-19 crisis, six questions were selected from absenteeism and presenteeism questions of the HPQ [8, 28], short version in Japanese: B3, B5a, B5c, B9, B10, and B11 (Table S2). HPQ is known as a self-reported instrumented to evaluate the workplace costs of health problems related to reduced job performance and absence [28]. In HPQ, absolute presenteeism score was calculated as the selected number on a 0 to 10 scale multiplied by 10 as directed in scoring rule, which made the score in the range of 0 to 100. The score of 0 represents the state of presenteeism with lowered work performance. The score of 40 or below would be considered as being at risk for future absence due to sickness [29]. Additionally, a relative presenteeism score was calculated according to the scoring rule of HPQ, where the range was restricted to 0.25 to 2.0, where a score of less than 1.0 represents a lower work performance of the responders compared to most workers in a similar job.

### Exposure variables

Factors which may affect presenteeism were taken from the fourth survey. It included the questions about social support, which was based on the six-item Japanese version of Social Support Questionnaire (SSQ) [30]. In this study, participants could answer the number of supporters in the range of 0 to more than 6 people, and the satisfaction level in the six-point Likert scale. The mean of six item were calculated for number of supporters and satisfaction levels separately. The original version of the SSQ was developed by Sarason et al. [31, 32]. To represent social interaction, three variables were selected: change in time spent talking with family, change in time spent talking with friends, and living with others or alone. The questions related to social interaction were asking the participants to rate the percentage of changes in current daily behavior using the unit of 10% between “−100%” to more than “+100%”, compared to the same month in the last year. We used five models for each exposure variable: model one for change in time spent talking with family, model two for change in time spent talking with friends, model three for living with others, model four for number of supporters, and model five for satisfaction for the social support. The number of supporters were categorized into three groups of no supporter, one to four supporters, or five and more supporters. The change in time spent on talking was categorized into three groups of increased, the same or decreased. The category of each variable is shown in the supplementary information (Table S3). Multiple linear regression was conducted with adjustment variables.

### Adjustment variables

We used a directed acyclic graph (DAG), which represented our hypothesis and the assumptions necessary for potential causal interpretation, to identify adjustment variables for each exposure variable. To select the variables entered in DAG, the factors related to presenteeism were summarized, as shown in Fig. S2, based on previous studies [4, 33, 34]. In this study, we focused on factors at the personal level. Figure S4 shows the DAG used to identify the adjustment sets. For the node called “social distancing”, a question was used that asked whether responders kept their physical distance from others more, the same or less compared to the time before pandemic. A question regarding the changes in hours of telework was applied for “Mode of work.” “Personality” was obtained from questions on “self-construal scale” [35].

## Results

**Fig. 1:**
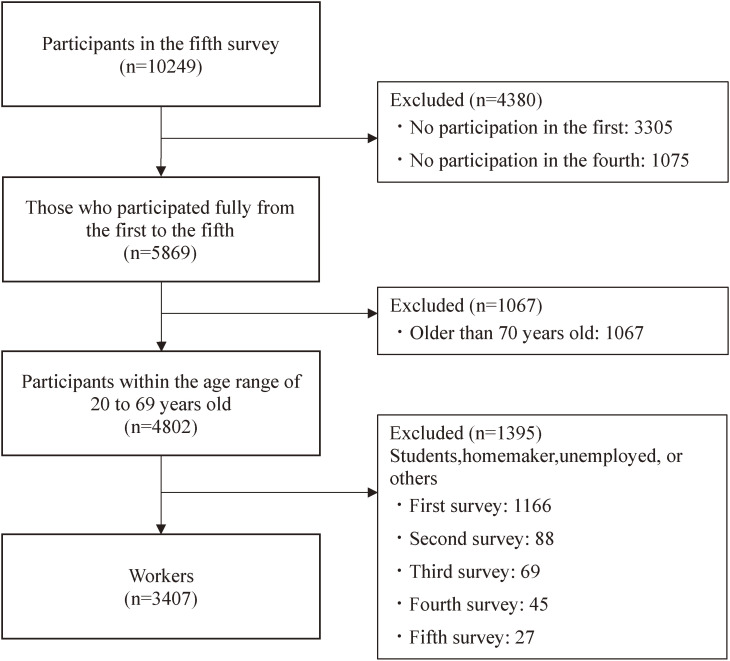
Flowchart of attrition

The 3,407 participants were included in this study. The attrition process is in Fig. 1. The demographics of the sample are shown in Table 1. The percentages of permanent workers was 49% (1676), whereas 25% (862) was temporary/part-time workers. The remaining 26% (869) were business owners/freelancers and technical/professional workers such as medical professionals. The supplementary information (Table S4) shows work-related characteristics of the participants. The median of working hours of all participants in the past seven days was 40 hours (IQR = 35–50).

**Table 1:** Characteristics of the study population

**Demographics**	**Overall**		**Age groups**									
			**20s**		**30s**		**40s**		**50s**		**60s**	
n	3407		494		706		826		825		556	
Sex (%)												
Women	1322	(39)	214	(43)	265	(38)	326	(40)	310	(38)	207	(37)
Men	2085	(61)	280	(57)	441	(62)	500	(60)	515	(62)	349	(63)
Income (%)												
<2000K	263	(8)	38	(8)	53	(8)	51	(6)	68	(8)	53	(10)
2000K∼6000K	1423	(42)	264	(53)	321	(46)	310	(38)	269	(33)	259	(47)
>=6000K	1220	(36)	130	(26)	261	(37)	326	(40)	359	(44)	144	(26)
NA	501	(15)	62	(13)	71	(10)	139	(17)	129	(16)	100	(18)
Education (%)												
Jr. High school	61	(2)	9	(2)	18	(3)	17	(2)	11	(1)	6	(1)
High school	994	(29)	114	(23)	168	(24)	248	(30)	280	(34)	184	(33)
Community Colledge	559	(16)	81	(16)	128	(18)	160	(19)	120	(15)	70	(13)
Undergraduate	1520	(45)	239	(48)	329	(47)	334	(40)	348	(42)	270	(49)
Graduate school	186	(6)	35	(7)	51	(7)	40	(5)	43	(5)	17	(3)
NA	87	(3)	16	(3)	12	(2)	27	(3)	23	(3)	9	(2)
Job Type (%)												
Permanent workers	1676	(49)	286	(58)	397	(56)	425	(52)	418	(51)	150	(27)
Temporary/Part-time workers	862	(25)	77	(16)	149	(21)	188	(23)	204	(25)	244	(44)
Technical/Professional workers	557	(16)	114	(23)	125	(18)	139	(17)	116	(14)	63	(11)
Business owners/Freelancers	312	(9)	17	(3)	35	(5)	74	(9)	87	(11)	99	(18)

**Fig. 2:**
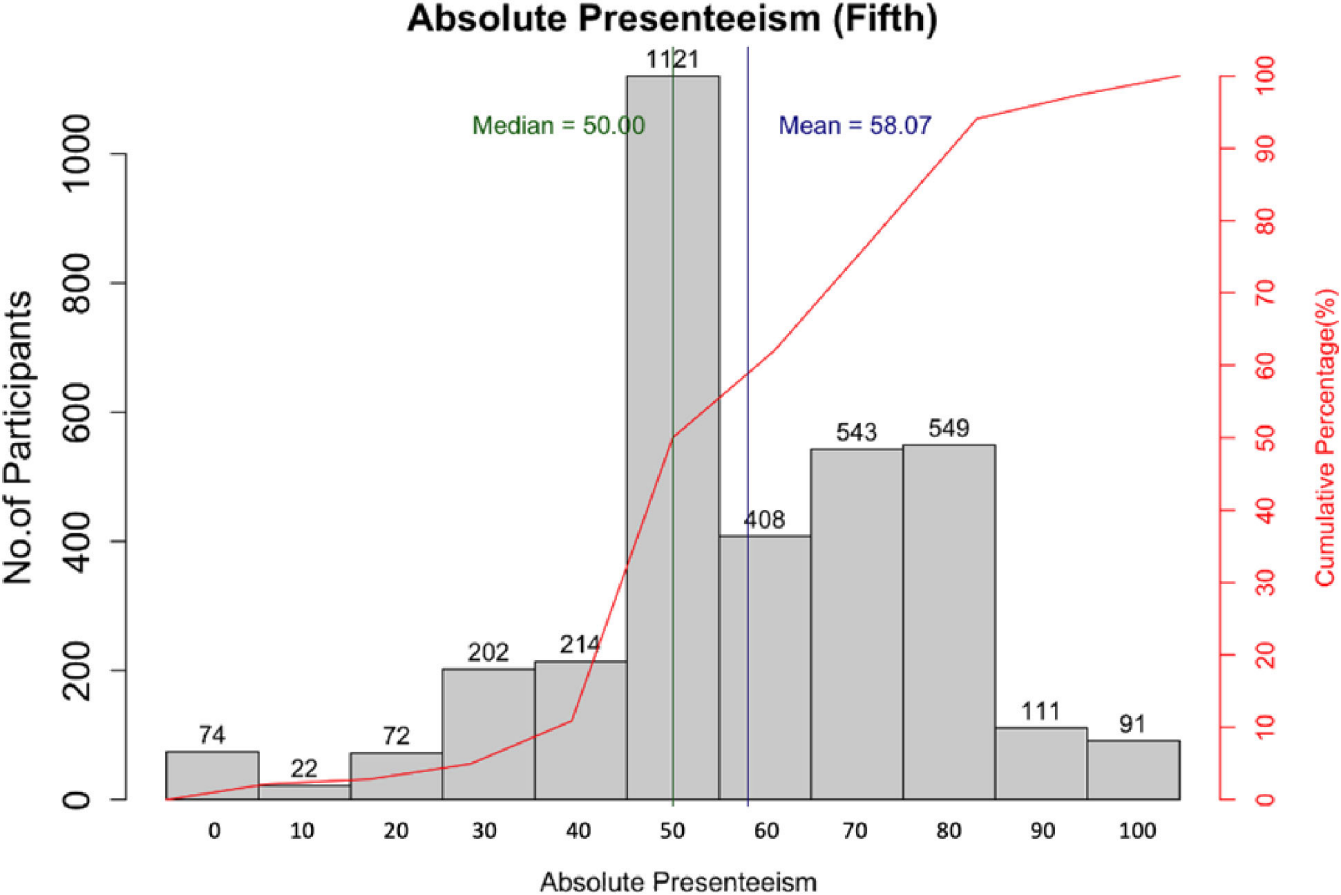
Distribution of absolute in the fifth survey

**Fig. 3:**
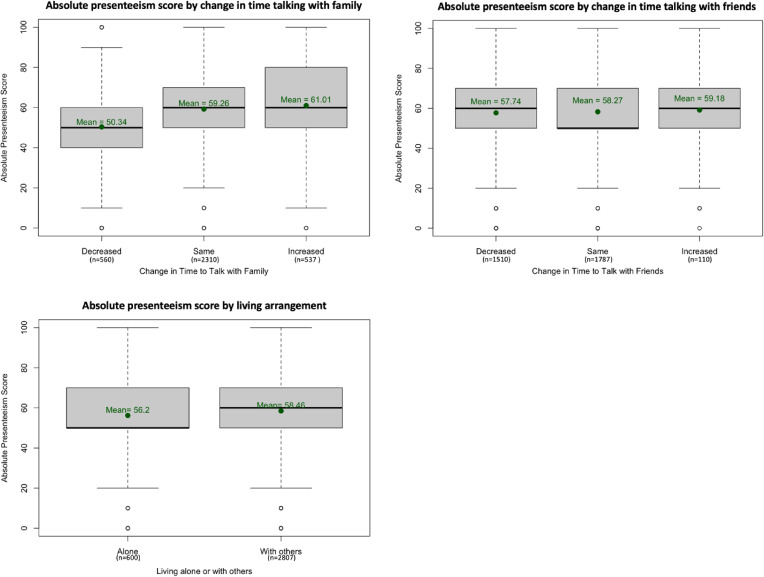
Social interaction and absolute presenteeism in the fifth survey

**Fig. 4:**
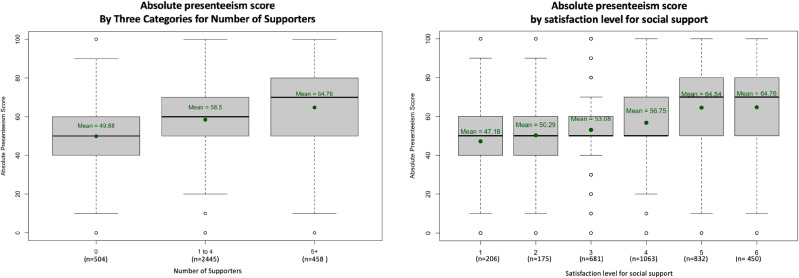
Social support and absolute presenteeism in the fifth survey

As the first part of the analysis, we described absolute presenteeism score and related factors. The score for all participants is displayed in Fig. 2. Some 33% (1121) participants scored their performance as 50, whereas 17% (584) participants scored it as 40 or below. The rest scored their performance as 60 or above. Additional information regarding participants who scored 40 or below in the first, the fourth and the fifth surveys is shown in Table S5. The relative presenteeism score is shown in Fig. S3. The relations between absolute presenteeism score and each factor of social interaction are shown in Fig. 3. Whereas 44% (1,510) of participants stated that the time spent talking with friends decreased, a smaller number of participants, 16% (560), answered “decreased” for the time spent talking with family. Some 18% (600) of participants were living alone, and 82% (2,807) of participants were living with others. The relations between absolute presenteeism score and each factor of social support are shown in Fig. 4. The scores tended to be higher for those who had more supporters. Similarly, the higher the reported satisfaction was, the higher the score of absolute presenteeism.

As the second part of the analysis, multiple linear regression analyses with a minimally sufficient adjustment set were conducted. As Table 2 shows, for social interaction, “change in time spent talking with family” was positively associated with absolute presenteeism score, whereas the “change in time spent talking with friends” and “living with others or alone” were not associated. For social support, both the number of supporters and the satisfaction were positively associated with absolute presenteeism score.

**Table 2:** Results of multiple regression models with outcome variable of absolute presenteeism and adjustment sets

**Models**	**Exposure variable**	**Categories of exposure variable**	**Regression coefficient**	**95% CI**		***P*-value**
				**2.5%**	**97.5%**	
1	Social interaction: Change in time spent talking with family	Decreased	Ref	Ref	Ref	Ref
		Same	7.733	5.328	10.138	<0.001
		Increased	8.070	5.423	10.717	<0.001

2	Social interaction: Change in time spent talking with friends	Decreased	Ref	Ref	Ref	Ref
		Same	−1.593	−3.219	0.033	0.055
		Increased	0.092	−3.703	3.887	0.962

3	Social interaction: Living with others	No	Ref	Ref	Ref	Ref
		Yes	0.657	−1.079	2.393	0.458

4	Social support: Number of supporters	0	Ref	Ref	Ref	Ref
		1 to 4	6.877	5.020	8.733	<0.001
		5 and more	12.980	10.531	15.429	<0.001

5	Social support: Satisfaction	1	Ref	Ref	Ref	Ref
		2	2.172	−1.570	5.915	0.255
		3	4.993	2.077	7.910	<0.001
		4	7.782	4.979	10.585	<0.001
		5	14.618	11.733	17.503	<0.001
		6	16.262	13.160	19.363	<0.001

Note: Variables of the minimally sufficient adjustment set for each model are shown below.

Model1. Demographic factors (age, sex, education, and household income), personality, change in time spent talking with friends, social distancing, work hours, and changes in hours of telework. Model2. Demographic factors, personality, change in time spent talking with family, social distancing, work hours, and changes in hours of telework. Model3. Demographic factors. Model 4 and 5. Demographic factors, personality, and living with others

## Discussion

This study found that the absolute presenteeism score among those who spent less time talking with family tended to be lower. In addition, the results of multiple regression indicated that there was a positive association between absolute presenteeism score and the change in time spent talking with family. However, the results of this study supported neither the associations between absolute presenteeism score and the change in time spent talking with friends nor the associations between absolute presenteeism score and living with others.

As family-work/work-family conflict has been known to be related to presenteeism [10], it seems to be natural that those who had more time to interact with family members may perform better at work in the “New Normal”. Although our study did not obtain information regarding the quality of interaction, considering how family can play an important role in individuals’ well-being by providing help to cope with stress [36], we believed that more time spent talking with family during the potentially stressful period caused by the pandemic was beneficial for workers.

On the other hand, a possible explanation for why the interaction with friends was not associated with absolute presenteeism score is the means of social interaction: how participants interacted with family and friends, such as in-person or virtual, might have affected the result. Previous studies have ununified views regarding the benefit of in-person and virtual interaction. A study reported that a higher number of partners for virtual interaction was associated with better mental health when in-person interaction was limited [37]. According to another study, both in-person and virtual interaction between households during the pandemic were associated with better mental health, even though the effect was limited in the case of virtual interaction [38]. By contrast, some reported that virtual interaction, which requires “close-up eye contact”, could be more stressful than in-person interaction [39]. Although the benefit of virtual interaction to mental health has been recognized, it seems to have some differences in comparison to in-person interaction. Therefore, the means of interaction were raised as a possible explanation.

With whom the individual shares accommodation and interacts might explain why the variable of living with others did not show any effect on presenteeism. Although the living arrangement in this study was summarized as living with someone or not, living with a spouse might have created a difference from living with someone other than a spouse [40]. Further investigation of living arrangements with a focus on the types of relationships may clarify the association between presenteeism and living arrangement.

This study also showed that both the greater number of supporters and higher satisfaction with the support were associated with higher absolute presenteeism score, which means less presenteeism during the pandemic. It is aligned with the results of previous studies in Japan [11, 22, 41]. Although we could not find a review article regarding presenteeism and social support, a study from China explored individual resilience with social support and job performance during the pandemic among healthcare professionals [22]. Their study reported that perceived social support positively affected an individual’s resilience, which may lead to better job performance [22]. A Japanese study before the pandemic reported that a perceived higher level of social support from superiors and co-workers had a protective effect on presenteeism [11]. Another study indicated that a perceived low level of social support from superiors, co-workers, family, and friends might not have a direct association, but might have an indirect association with presenteeism, by mediating psychological and physical stress responses and sleep disturbance [41]. Our result was also in accordance with our anticipation based on the reports that rewarding social interaction and support may prevent psychopathologies with their positive impacts on individual’s physical and emotional well-being [23].

Whereas previous studies regarding presenteeism and social support tended to focus on specific workplaces, this study did not specify the job types of participants. Additionally, the questions about social support were asked in a more general sense without limiting the questions to the workplace. Therefore, the unique finding of this study was that the social support in general reduced the presenteeism of workers in various occupations in the “new normal”. An investigation on how each dimension of social support, such as the reception or provision of support, and instrumental or emotional support may affect presenteeism is a subject for future research. Potential for practical implications would be to expand the availability of work-life balance education programs or the employee assistance program for social life. Because suitable assistance may differ depending on the job types, interventions for specific job types may be useful for future study. Moreover, it is important to inform policymakers that presenteeism should be accounted into the possible social cost upon the drastic action such as social distancing and lock down.

This study had several limitations. First, the participants were online panelists, who might have unique characteristics such as a higher digital literacy compared with the general population. Therefore, generalizability may be limited. However, because the target population did not include older adults, but those aged between 20 to 69 years, the concerns regarding digital literacy may not be as significant. This research also achieved the objective of obtaining participants from all prefectures in Japan and from various age groups. Moreover, the unique point of our study was that presenteeism was repeatedly measured for a wide range of age groups without being limited by specific types of jobs during COVID-19 pandemic in Japan. Second, the regression analyses were conducted with the minimally sufficient adjustment sets that we selected based on the knowledge gained from previous research; and this selection may reflect our interpretation of evidence in previous research. We also note that our survey could not include full HPQ due to the page limitations. Nonetheless, the strength of this study was that we could adjust confounders to avoid multicollinearity benefiting from the comprehensive nature of our survey.

In conclusion, our results suggested that social support reduced the presenteeism of the workers in Japan during the “new normal” due to COVID-19 pandemic. Social interaction with family also relieved presenteeism.

## Supplementary information

**Additional file 1:** **Table S1** A summary of excerpts from “example of practicing the ‘new normal’” in Japan [2]. **Table S2** A list of questions from WHO HPQ that were used in our study. **Table S3** Units of exposure variables for the regressions analysis. **Table S4** Work-related characteristics of the study population. **Table S5.** Participants who scored 40 or below in absolute presenteeism by age groups.

**Additional file 2:** **Figure S1** Timings of the surveys and number of new COVID-19 cases in Japan. **Figure S2** Factors related to presenteeism based on previous studies (4, 31, 32). **Figure S3** Distribution of relative presenteeism in the fifth survey. **Figure S4** Simplified version of directed acyclic graph used for the analysis.

## Declarations

### Availability of data and materials

Data used in this study are not open to the public.
