# Supplementary material for: Presenteeism and social interaction in the “new normal” in Japan: a longitudinal questionnaire study
Source: Environ Health Prev Med. 2024 Jan 20;29:3. doi: 10.1265/ehpm.23-00201 (PMC10808005; doi:10.1265/ehpm.23-00201)
Supplement: Supplementary file 1 — Additional file 1: Table S1 A summary of excerpts from “example of practicing the ‘new normal’” in Japan [2]. Table S2 A list of questions from WHO HPQ that were used in our study. Table S3 Units of exposure variables for the regressions analysis. Table S4 Work-related characteristics of the study population. Table S5. Participants who scored 40 or below in absolute presenteeism by age groups. [file ehpm-29-003-s001.docx]

| Table S1 A summary of excerpts from “example of practicing the ‘new normal’” in Japan (2) | | |
| --- | --- | --- |
| Four domains | | Examples |
| 1 | Basic infection  prevention measure | Keeping physical distance |
|  |  | Wearing a mask |
|  |  | Washing hands |
|  |  | Refrain from traveling to and from where the infection is prevailing |
| 2 | Basic lifestyle for daily life | Avoid gatherings in crowded places, close contact settings and closed spaces |
| 3 | Lifestyle for each scene of daily life | Use online shopping, electric payment |
|  |  | Refrain from chatting in public transports and during meals |
|  |  | Avoid banquets or meetings with large numbers (Family ceremonial occasions) |
| 4 | New working style | Work remotely |
|  |  | Use online meetings |
|  |  | Exchange business cards online |

Table S2 A list of questions from WHO HPQ that were used in our study.

| Items | | Questions |
| --- | --- | --- |
|  | B3 | About how many hours altogether did you work in the past 7 days? |
|  | B5 | Now please think of your work experiences over the past 4 weeks (28 days). In the spaces provided below, write the number of days you spent in each of the following work situations. In the past 4 weeks (28 days), how many days did you... |
|  | B5a | ..missanentireworkdaybecauseofproblemswithyourphysicalormental health? (Please include only days missed for your own health, not someone else’s health.) |
|  | B5c | ...misspartofaworkdaybecauseofproblemswithyourphysicalormental health? (Please include only days missed for your own health, not someone else’s health.) |
|  | B9 | On a scale from 0 to 10 where 0 is the worst job performance anyone could have at your job and 10 is the performance of a top worker, how would you rate the usual performance of most workers in a job similar to yours? |
|  | B10 | Using the same 0-to-10 scale, how would you rate your usual job performance over the past year or two? |
|  | B11 | Using the same 0-to-10 scale, how would you rate your overall job performance on the days you worked during the past 4 weeks (28 days)? |

| Table S3 Units of exposure variables for the regressions analysis | | | |  |
| --- | --- | --- | --- | --- |
| Model | Category of exposure variable | Exposure variable | Categories of variable |  |
|  |  |  |  |  |
| 1 | Social interaction | Change in time spent talking with family | Decreased= -1, Same=0, Increased=1 |  |
| 2 |  | Change in time spent talking with friends | Decreased= -1, Same=0, Increased=1 |  |
| 3 |  | Living with others | Living alone=0, Living with others=1 |  |
| 4 | Social support | Mean number of supporters | 0-0.4=0, 0.5-4.4=1, More than 4.4=2 |  |
| 5 |  | Mean level of satisfaction | 1-1.4=1, 1.5-2.4=2, 2.5-3.4=3,  3.5-4.4=4, 4.5-5.4=5, More than 5.4=6 |  |

| Table S4 Work-related characteristics of the study population | | | | | | |
| --- | --- | --- | --- | --- | --- | --- |
| Work related characteristics | Overall | Age groups | | | | |
|  |  | 20s | 30s | 40s | 50s | 60s |
| Total work hours in past 7 days (median [IQR]) | 40.00 [35.00, 50.00] | 40.00 [40.00, 50.00] | 40.00 [36.00, 50.00] | 40.00 [36.25, 50.00] | 40.00 [35.00, 50.00] | 35.00 [20.00, 40.00] |
| Absolute Presenteeism Score for past 28 days (mean (SD)) | 58.07 (19.71) | 54.09 (20.17) | 54.53 (19.24) | 56.72 (19.71) | 60.84 (18.91) | 63.97 (19.09) |

| Table S5. |  |  |  |  |  |  |  |  |  |  |  |  |
| --- | --- | --- | --- | --- | --- | --- | --- | --- | --- | --- | --- | --- |
| Participants who scored 40 or below in absolute presenteeism by age groups | | | | | | | | | | | | |
|  | 1st survey | | | | 4th survey | | | | 5th survey | | | |
| Age Groups (n) | n |  | (%) |  | n |  | (%) |  | n |  | (%) |  |
| All (3407) | 437 | ( | 13 | ) | 598 | ( | 18 | ) | 584 | ( | 17 | ) |
| 20s (494) | 88 | ( | 18 | ) | 121 | ( | 24 | ) | 123 | ( | 25 | ) |
| 30s (706) | 114 | ( | 16 | ) | 164 | ( | 23 | ) | 152 | ( | 22 | ) |
| 40s (826) | 97 | ( | 12 | ) | 156 | ( | 19 | ) | 154 | ( | 19 | ) |
| 50s (825) | 89 | ( | 11 | ) | 109 | ( | 13 | ) | 111 | ( | 13 | ) |
| 60s (556) | 49 | ( | 9 | ) | 48 | ( | 9 | ) | 44 | ( | 8 | ) |
