# Supplementary material for: Presenteeism and social interaction in the “new normal” in Japan: a longitudinal questionnaire study
Source: Environ Health Prev Med. 2024 Jan 20;29:3. doi: 10.1265/ehpm.23-00201 (PMC10808005; doi:10.1265/ehpm.23-00201)

Supplementary figures

Figure S1 Timings of the surveys and number of new COVID-19 cases in Japan


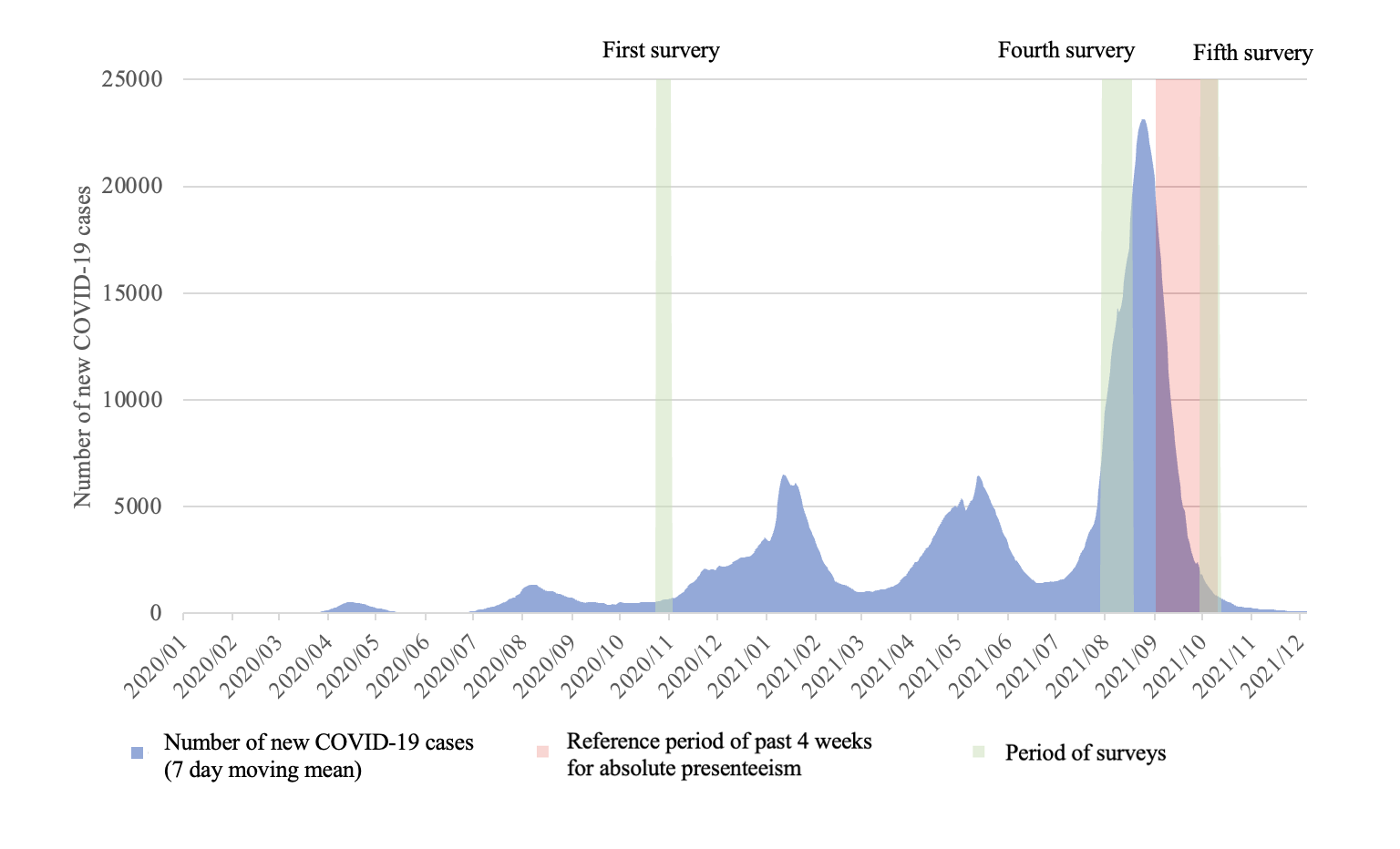


Figure S2 Factors related to presenteeism based on previous studies (4, 31, 32)


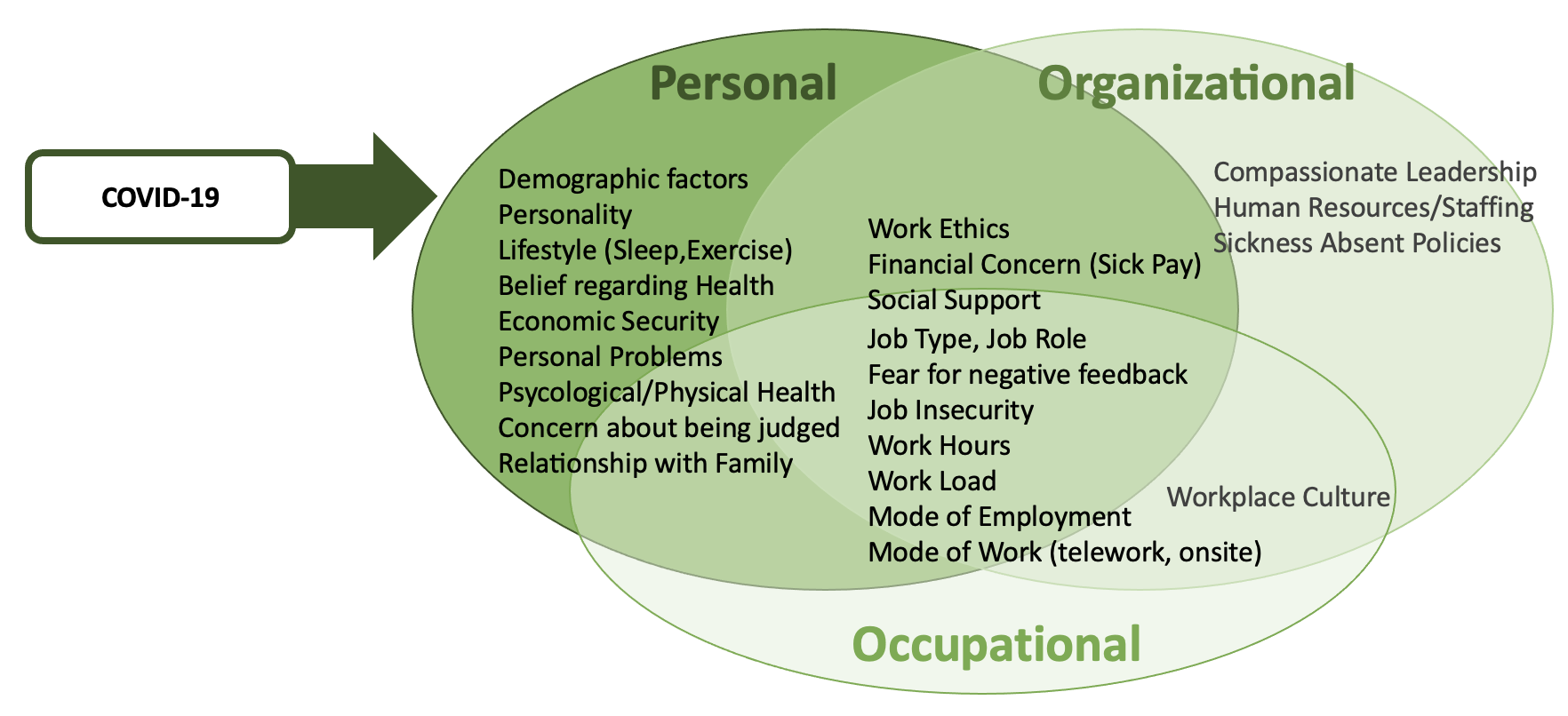


Figure S3 Distribution of relative presenteeism in the fifth survey


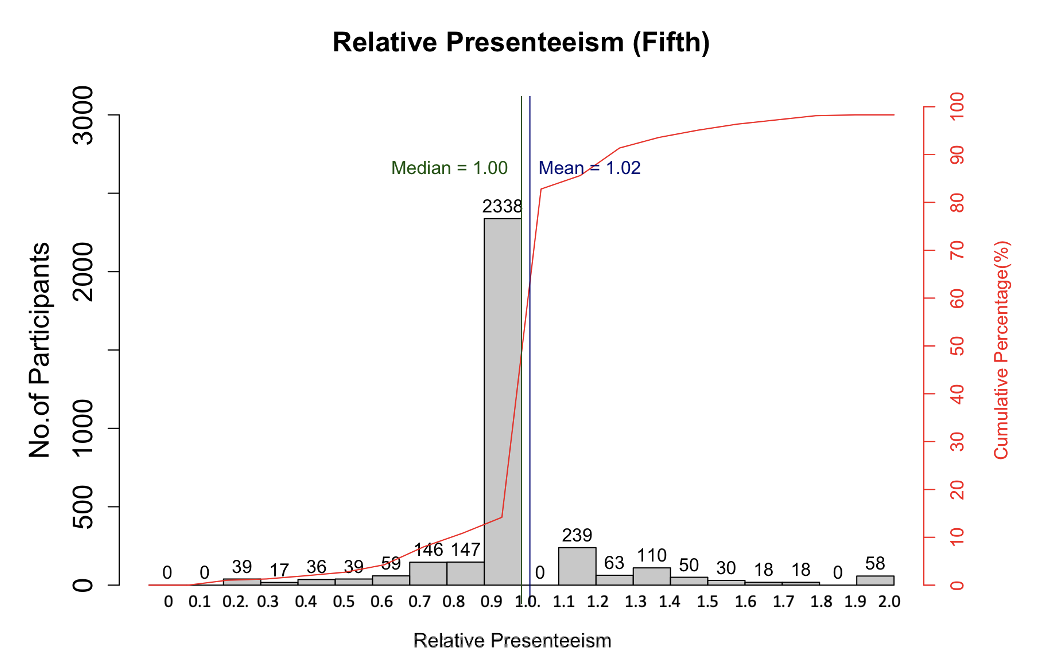


Figure S4 Simplified version of directed acyclic graph used for the analysis.


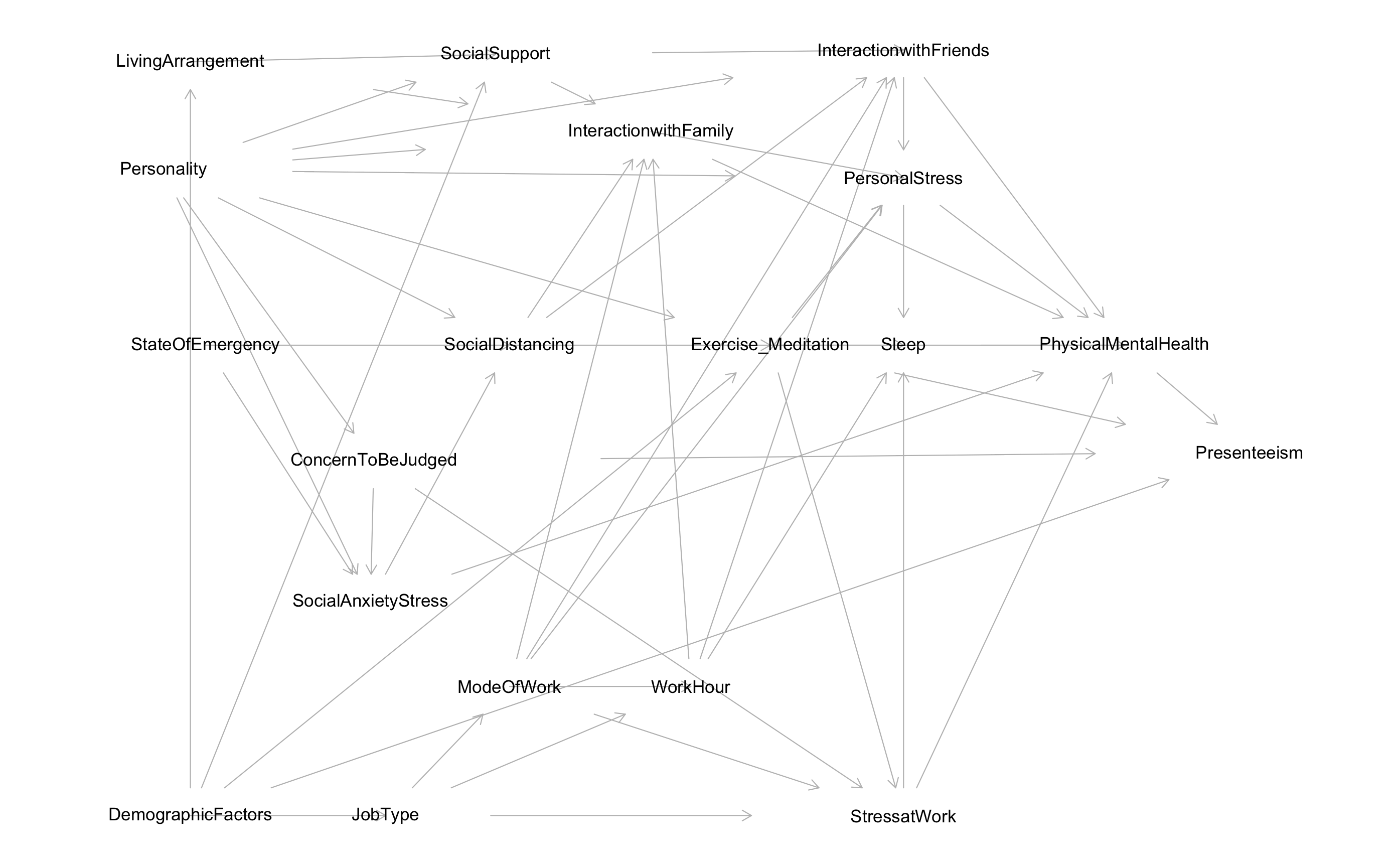

Supplement: Supplementary file 2 — Additional file 2: Figure S1 Timings of the surveys and number of new COVID-19 cases in Japan. Figure S2 Factors related to presenteeism based on previous studies (4, 31, 32). Figure S3 Distribution of relative presenteeism in the fifth survey. Figure S4 Simplified version of directed acyclic graph used for the analysis. [file ehpm-29-003-s002.docx]
